# Photoplethysmography for blood volumes and oxygenation changes during intermittent vascular occlusions

**DOI:** 10.1007/s10877-017-0030-2

**Published:** 2017-05-25

**Authors:** T. Y. Abay, P. A. Kyriacou

**Affiliations:** School of Mathematics, Computer Sciences and Engineering, City, University of London, London, UK

**Keywords:** Photoplethysmography, Vascular occlusions, Near infrared spectroscopy, Blood volumes, Oxygenation

## Abstract

Photoplethysmography (PPG) is an optical technique that measures blood volume variations. The main application of dual-wavelength PPG is pulse oximetry, in which the arterial oxygen saturation (SpO$_2$) is calculated noninvasively. However, the PPG waveform contains other significant physiological information that can be used in conjunction to SpO$_2$ for the assessment of oxygenation and blood volumes changes. This paper investigates the use of near infrared spectroscopy (NIRS) processing techniques for extracting relative concentration changes of oxygenated ($\Delta$HbO$_2$), reduced ($\Delta$HHb) and total haemoglobin ($\Delta$tHb) from dual-wavelength PPG signals during intermittent pressure-increasing vascular occlusions. A reflectance PPG sensor was attached on the left forearm of nineteen (n = 19) volunteers, along with a reference NIRS sensor positioned on the same forearm, above the left brachioradialis. The investigation protocol consisted of seven intermittent and pressure-increasing vascular occlusions. Relative changes in haemoglobin concentrations were obtained by applying the modified Beer–Lambert law to PPG signals, while oxygenation changes were estimated by the difference between red and infrared attenuations of DC PPGs (A$_{Ox}$ = $\Delta$A$_{IR}$ − $\Delta$A$_R$) and by the conventional SpO$_2$. The $\Delta$HbO$_2$, $\Delta$HHb, $\Delta$tHb from the PPG signals indicated significant changes in perfusion induced by either partial and complete occlusions (p < 0.05). The trends in the variables extracted from PPG showed good correlation with the same parameters measured by the reference NIRS monitor. Bland and Altman analysis of agreement between PPG and NIRS showed underestimation of the magnitude of changes by the PPG. A$_{Ox}$ indicated significant changes for occlusion pressures exceeding 20 mmHg (p < 0.05) and correlation with tissue oxygenation changes measured by NIRS, while SpO$_2$ had significant changes after 40 mmHg (p < 0.05). Relative changes in haemoglobin concentrations can be estimated from PPG signals and they showed a good level of accuracy in the detection of perfusion and oxygenation changes induced by different degrees of intermittent vascular occlusions. These results can open up to new applications of the PPG waveform in the detection of blood volumes and oxygenation changes.

## Introduction

Monitoring regional oxygenation and blood volumes changes in tissues non-invasively is vital in clinical practice. This motivated researchers and leading manufacturers in developing new medical instrumentation and measurement methods that could provide physiological information for guiding therapy or improving patient’s clinical outcomes [1, 2]. One of the most commonly used techniques to assess regional oxygenation and blood volumes non-invasively is near infrared spectroscopy (NIRS) [3–5]. The technique is based on the assumption that near infrared light, due to low absorption and high scattering properties, penetrates deep into living tissues and is attenuated in proportion to the changes in the concentration of chromophores in the tissue [3–6]. In NIRS, the modified Beer–Lambert law (MBLL) is applied to light attenuations for the estimation of relative changes in the concentration of oxygenated ($\Delta$HbO$_2$), reduced ($\Delta$HHb), and total haemoglobin ($\Delta$tHb), assuming that HbO$_2$ and HHb are the only chromophores changing concentration during the measurement [4]. Even though the estimated $\Delta$HbO$_2$, $\Delta$HHb, and $\Delta$tHb are usually not absolute but relative to baseline, their trends can still provide an indication of changes in arterial oxygen content ($\Delta$HbO$_2$), venous blood ($\Delta$HHb), and overall blood volume ($\Delta$tHb) [3, 5]. Absolute concentrations of oxygenated, reduced, and total haemoglobin can also be quantified by more complex NIRS measurement techniques such as time-resolved spectroscopy or phase-resolved spectroscopy [3]. If the absolute concentrations of the three haemoglobins can be determined, a tissue oxygenation index (also known as TOI, rSO$_2$, or StO$_2$) can be calculated from the ratio of HbO$_2$ and tHb [3, 5]. Another approach, known as spatially resolved spectroscopy, allows the calculation of the tissue oxygenation index by computing optical signals acquired from two photodetectors positioned closed to each other [3, 7].

Another technique used to monitor blood volumes is photoplethysmography (PPG). PPG is an optic-based technique, which exploits the changes in light absorption of living tissue during the cardiac cycle [8–10]. Light is shone in tissues and the detected light intensity forms an electric signal known as Photoplethysmograph [9]. The PPG signal is composed of a pulsatile component (AC) and a relatively slow varying component (DC). The AC component is related to the pulsating arteries and arterioles, whereas the DC represents the constant absorption of non-pulsatile tissue within the light path (i.e. venous blood, venoules, non-pulsatile arterial blood, etc.) [9]. The main application of PPG is in pulse oximetry, where the arterial oxygen saturation (SpO$_2$) is estimated from the ratio of both AC and DC PPG components at red and infrared wavelengths [8, 9].

In their principle of operation, PPG and NIRS are two similar techniques, with a key difference in how the acquired signals are processed for estimating physiological parameters. While the PPG signals at red and infrared wavelengths are typically used for the estimation of arterial SpO$_2$ and heart rate, NIRS provides an indication of mixed saturation of the sampled volume (arterial, capillaries, and venous). For this, NIRS could be considered superior in assessing changes in both incoming blood oxygen content (arterial blood) and oxygen consumption (venous blood) [1, 3–5]. PPG usually provides blood volume information by focusing just on the pulsatile AC PPG component [8], whereas NIRS excludes the pulsatile nature of arterial blood and expresses changes in blood volume and oxygenation by relative changes in haemoglobin concentrations [4]. The two techniques have also different abilities in penetrating into tissue. Reflectance PPG is believed to penetrate only a few millimetres [11], whereas NIRS can penetrate up to two centimetres, depending on the inter-optodes separation distance [3, 5, 12]. This greater penetration depth is however compromised with a bulkier geometry of NIRS sensors (i.e. emitter-detector distance usually >2.5 cm), hence limiting their application only to large surface areas. On the other side, PPG sensors can be easily miniaturised to a few millimetres, with the capability of acquiring the signals from a multitude of locations on the body such as fingers, earlobes, forehead, ear canal, oesophagus, bowel, etc [9, 13–15]. Finally, NIRS devices are commonly expensive [16], and thus not used as routinely as pulse oximeters.

Based on the similar operation and technology, it should be possible to apply the MBLL to the DC PPG signals acquired from a pulse oximeter sensor for estimating relative changes in haemoglobin concentrations, as in NIRS. By doing so, the PPG signals can be used to simultaneously assess more physiological parameters in addition to SpO$_2$, heart rate, and perfusion index, normally estimated by pulse oximeters. In a previous publication, we demonstrated the feasibility of using this method for obtaining perfusion information from dual-wavelength PPG signals [17]. This paper aims to investigate further the capability and responsivity of PPG signals in indicating perfusion and oxygenation changes induced by different degrees of intermittent vascular occlusions in the forearm, either of venous or arterial nature. The changes in the parameters derived from PPG were compared with a reference NIRS monitor by means of correlation and agreement analysis.

## Methods

### Instrumentation

In the investigation carried out, a research PPG system (ZenPPG, City, University of London, London, UK) was used to acquire raw PPG signals (AC + DC) from the forearm. ZenPPG is a modular, dual-wavelength and dual channel research PPG acquisition system, which is capable of intermittent switching of light sources, the sampling of red/infrared signals and output of the signals to data acquisition systems [18]. By acquiring dual-wavelength raw PPG signals, the device can also function as a research pulse oximeter.

A reflectance PPG sensor was employed for the acquisition of PPG signals from the forearm. The sensor comprised of two red LEDs (660 nm) and two infrared LEDs (880 nm). When connected to the PPG processing system, the two wavelengths were driven and sequentially switched ON/OFF for independent sampling of dual-wavelength PPGs. A silicon photodiode with a large active area (7.5 mm$^2$) was employed for the detection of light. All the LEDs were positioned at a separation distance of 5 mm from the photodiode.

**Fig. 1:**
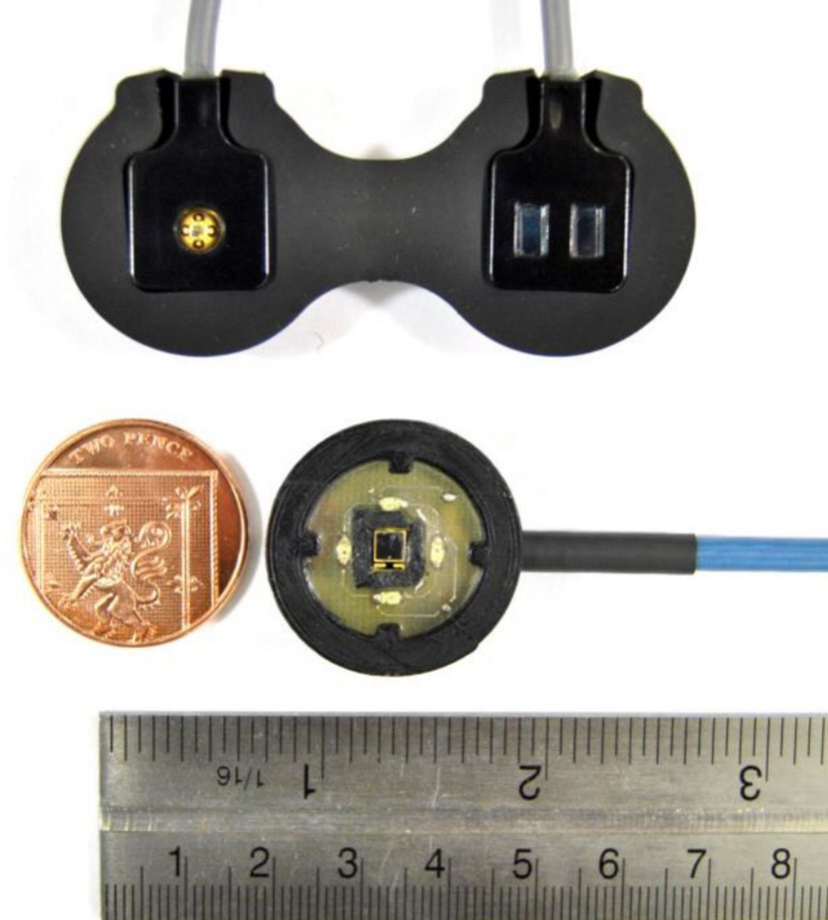
Developed reflectance PPG sensor (*bottom*) and commercial NIRS sensor (*top*)

A commercial NIRS monitor (NIRO 200NX, Hamamatsu Photonics, Hamamatsu, Japan) was used for the measurement of relative changes in haemoglobin concentration ($\Delta$HbO$_2$, $\Delta$HHb, and $\Delta$tHb) and oxygenation (TOI) from the forearm. The monitor shines light at three wavelengths (735, 810, and 850 nm), while two silicon photodiodes, placed few millimetres from each other, are used for light detection (see Fig. 1). The light intensities from one of the photodiodes are used along with the MBLL for the estimation of changes in $\Delta$HbO$_2$, $\Delta$HHb, and $\Delta$tHb, while the slopes of the light attenuations at the three wavelengths between the two photodiodes outputs are simultaneously processed by the spatially resolved spectroscopy in order to estimate k $\cdot$ HbO$_2$ and k $\cdot$ HHb, where the constant k represents the scattering properties of the tissue in the near infrared region [7]. The Tissue Oxygenation Index (TOI) is then calculated from the ratio TOI = k $\cdot$ HbO$_2$/(k $\cdot$ HbO$_2$ + k $\cdot$ HHb) [7]. In this study, an NIRS sensor with 4 cm separation distance between light source and detector was employed for the measurements. Figure 1 shows both the reflectance PPG sensor and the NIRS sensor that were employed in this study.

### Investigation protocol

In order to investigate the ability to monitor relative changes in blood volumes and oxygenation using photoplethysmography, an investigation on healthy volunteers was carried out in the Biomedical Engineering research laboratories at City, University of London. Ethical approval was obtained from the Senate Research Ethics Committee at City, University of London and written informed consent was obtained from all individual participants included in the study.

Nineteen (19) healthy subjects [median age: 30 (IQR = 26–34); 13 males and 6 females] were recruited for the investigation. The subjects were accommodated in a comfortable chair with their left arm rested on a soft cushion. The experiment was carried out in a room maintained at a constant ambient temperature of 23 ± 1 °C. Prior to the positioning of the sensors, the volunteer’s blood pressure was measured by an automatic blood pressure monitor (HEM-907, Omron Healthcare Co. Ltd, Kyoto, Japan). A sphygmomanometer, comprising of a cuff and a bulb (Big Ben Round, Rudolf Riester GmbH, Jungingen, Germany), was used for inducing vascular occlusions on the forearm. The cuff was placed around the upper left arm and occlusions were performed by manually inflating the cuff through the bulb. The NIRS sensor was placed above the left brachioradialis, whereas the reflectance PPG sensor was placed on the same area of the forearm, but distal with respect to the NIRS sensor. Both sensors were attached to the skin by double-sided clear medical adhesive tape and no pressure was applied to the sensors throughout the measurements.

A vascular occlusion protocol (VOP) was performed in order to induce changes in perfusion in the measurement area. The VOP consisted of seven intermittent occlusions at 20, 40, 60, 80, and 100 mmHg, followed by occlusions at the volunteer’s systolic pressure and at 20 mmHg over the subject’s systolic pressure (total occlusion). Each occlusion lasted for one minute and it was followed by a one-minute recovery, allowing a resting period between the occlusions.

### Data processing, analysis and statistics

All signals were simultaneously acquired by data acquisition cards at a sampling frequency of 1 kHz on LabVIEW (National Instrument, Austin, TX, USA). The acquisition program was developed for displaying and saving of the measurements in real time on a PC. $\Delta$HbO$_2$, $\Delta$HHb, $\Delta$tHb, and TOI acquired from the NIRS monitor were low-pass filtered at 0.1 Hz. The AC PPGs were obtained by band-pass filtering the raw PPG signals (band-pass: 0.5–5 Hz), whereas the DC PPGs were obtained by low-pass filtering the raw signals at 0.1 Hz. Relative changes in haemoglobin concentrations (i.e. $\Delta$HbO$_2$, $\Delta$HHb, and $\Delta$tHb) were estimated by solving the MBLL for dual-wavelength DC PPG signals, as shown in Eqs. 1–3 [17].

1 $$\begin{aligned} \Delta [HbO_2]~=~\frac{\Delta A_R \cdot \alpha _{IR_{HHb}} - \Delta A_{IR} \cdot \alpha _{R_{HHb}}}{\alpha _{R_{HbO_2}} \cdot \alpha _{IR_{HHb}} - \alpha _{IR_{HbO_2}} \cdot \alpha _{R_{HHb}}} \end{aligned}$$

2 $$\begin{aligned} \Delta [HHb]~=~\frac{\Delta A_{IR} \cdot \alpha _{R_{HbO_2}} - \Delta A_{R} \cdot \alpha _{IR_{HbO_2}}}{\alpha _{R_{HbO_2}} \cdot \alpha _{IR_{HHb}} - \alpha _{IR_{HbO_2}} \cdot \alpha _{R_{HHb}}} \end{aligned}$$

3 $$\begin{aligned} \Delta [tHb]~=~\Delta [HbO_2] + \Delta [HHb] \end{aligned}$$

where $\Delta$AR and $\Delta$AIR are the changes from baseline in DC PPG attenuations (or optical densities) at red and infrared wavelength respectively, and $\alpha$ are the millimolar absorptivity coefficients of oxygenated (HbO$_2$) and reduced haemoglobin (HHb) at red (R) and infrared (IR) wavelengths. Since the effective pathlength travelled by the light was unknown, the relative changes in concentrations were expressed in mM cm (concentration $\times$ pathlength). The $\Delta$tHb was calculated as the sum of $\Delta$HbO$_2$ and $\Delta$HHb, assuming that these were the only chromophores varying throughout the measurement. The DC PPGs were also used for estimating the changes in oxygenation in the measurement area. Changes in oxygenation were calculated by determining the index A$_{Ox}$ = [($\Delta$A$_{IR}$ − $\Delta$A$_R$) $\times$ 100%], with A$_{IR}$ and A$_R$ being respectively the changes in light attenuations at infrared and red from the baseline, thus A$_{Ox}$ expressing the percentage changes in oxygenation from the baseline. The arterial oxygen saturation (SpO$_2$) was calculated from red and infrared PPG signals by applying an empirical equation relating the ratio of the AC and DC component at both wavelengths and the SpO$_2$ [9].

The results are presented as median and interquartile ranges (IQR = Q$_3$ − Q$_1$), with Q$_3$ and Q$_1$ being the upper (75%) and the lower (25%) percentile. Median values were obtained by averaging the measurements over thirty seconds of baseline readings and over the last thirty seconds of each occlusion. The changes in the haemodynamic parameters induced by occlusions were assessed by using a non-parametric test (Wilcoxon signed rank test). Significant changes during each stage of the protocol were verified by paired tests between mean values in baseline and during interventions. A p value <0.05 was considered statistically significant. Trends in relative changes in haemoglobin concentrations estimated from the PPGs were compared to the trends of the same NIRS measurements by assessing correlation and computing the Pearson’s correlation coefficient *r* and the 95% confidence intervals (CI). All data and statistical analysis were performed in MatlabR2013 (The MathWorks Inc., USA).

## Results

**Fig. 2:**
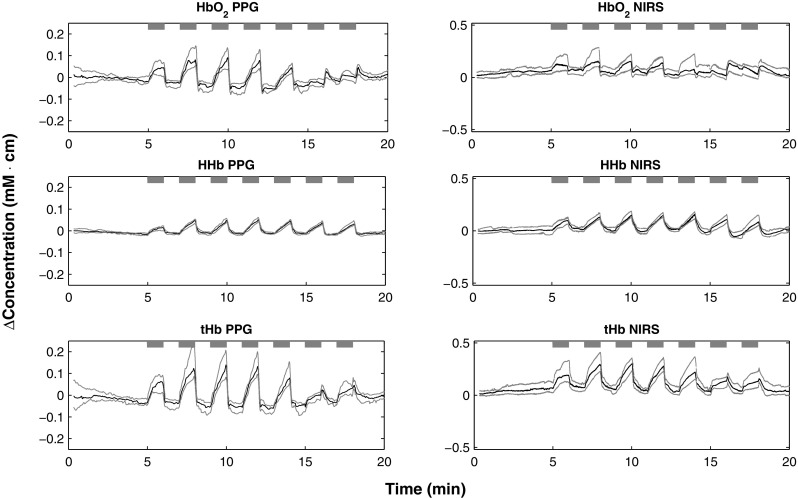
Changes in relative haemoglobin concentrations estimated by both PPG and NIRS during the protocol across all the volunteers.* Black* traces are the medians, while the* grey* traces represent the upper (75%) and lower (25%) quartiles. $\Delta$HbO$_2$: oxygenated haemoglobin, $\Delta$HHb: reduced haemoglobin, $\Delta$tHb: total haemoglobin.* Grey horizontal bars *indicate the duration of the occlusions. The order of the seven occlusions was 20, 40, 60, 80, 100 mmHg, systolic pressure, and over-systolic pressure. Each occlusion lasted for 1 min and it was followed by one-minute recovery

Figure 2 illustrates the medians, upper, and lower quartiles of the relative concentrations of oxygenated, reduced, and total haemoglobin estimated by the PPG during the protocol across all the subjects. For comparison, the same figure shows the same parameters estimated by NIRS. The PPG-derived parameters were able to indicate changes in blood volume and oxygenation throughout the occlusion protocol. The $\Delta$HbO$_2$, $\Delta$HHb, and $\Delta$tHb from PPG had significant changes in all occlusions (p < 0.05), except for $\Delta$HbO$_2$ during over-systolic occlusion (p = 0.947) and for $\Delta$tHb during systolic occlusion (p = 0.810). Similarly, NIRS-derived parameters had significant changes (p < 0.05) during all occlusions, except for $\Delta$HbO$_2$ during systolic (p = 0.266) and over-systolic occlusions (p = 0.499).

**Fig. 3:**
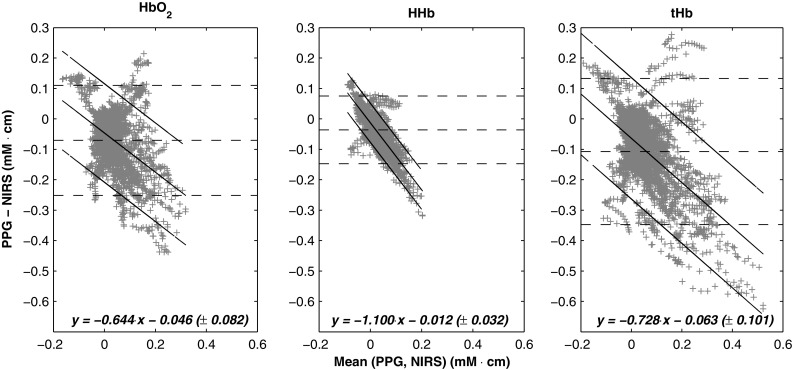
Bland and Altman analysis of the haemoglobin concentrations changes estimated from PPG and NIRS. X-axes are the mean between the measures, while y-axes are the differences. The* horizontal dotted line* indicates the standard limits of agreement between the measures (mean bias ± 1.96 $\times$ SD), while the* black solid line*s represent the fitted limits of agreement, with the regression line on the data and the 95% confidence intervals. The equations of the* fitted lines* are presented at the* bottom* of each plot. HbO$_2$: oxygenated haemoglobin, HHb: reduced haemoglobin, tHb: total haemoglobin. For each plot, a total number of points n = 3222 were used

Quantitative analysis between the relative concentrations estimated from the PPG and the NIRS was performed by Bland and Altman plots. Figure 3 shows the Bland and Altman plots between the variables estimated, in which the limits of agreement between PPG and NIRS were $\textendash$0.070 ± 1.96 $\times$ 0.092, $\textendash$0.036 ± 1.96 $\times$ 0.056, and $\textendash$0.107 ± 1.96 $\times$ 0.122 for $\Delta$HbO$_2$, $\Delta$HHb, and $\Delta$tHb respectively. The relationship between differences and means however varied with the magnitude of the change and regression lines with the 95% CI were fitted on the data to express this relationship. The equations of the fitted lines are shown in Fig. 3, revealing a consistent underestimation of $\Delta$HbO$_2$, $\Delta$HHb, and $\Delta$tHb from PPG when compared to NIRS.

In order to assess the trending capability of PPG compared to NIRS, correlation analysis was carried out between the relative haemoglobin concentrations estimated by PPG and NIRS. Table 1 shows the mean correlation coefficients *r* calculated between PPG and NIRS measurements across all steps of the protocol. The parameters showed high correlation in all occlusions, except HbO$_2$ during over-systolic occlusion with a correlation coefficient *r* = 0.35 (−0.08, 0.71).

**Table 1:** Pearson’s correlation coefficients *r* between relative changes in concentration of reduced ($\Delta$HHb), oxygenated ($\Delta$HbO_2_), and total haemoglobin ($\Delta$tHb) measured by PPG and NIRS during the protocol

Occlusions	Correlation coefficient *r*		
	HbO$_2$	HHb	tHb
20 mmHg	0.89 (0.68, 0.94)	0.89 (0.72, 0.95)	0.93 (0.81, 0.97)
40 mmHg	0.92 (0.76, 0.96)	0.94 (0.88, 0.98)	0.97 (0.92, 0.98)
60 mmHg	0.90 (0.78, 0.96)	0.96 (0.89, 0.98)	0.96 (0.91, 0.98)
80 mmHg	0.93 (0.81, 0.97)	0.97 (0.91, 0.98)	0.97 (0.92, 0.98)
100 mmHg	0.85 (0.65, 0.94)	0.97 (0.91, 0.98)	0.96 (0.91, 0.98)
Systolic	0.59 (0.10, 0.79)	0.92 (0.76, 0.96)	0.80 (0.63, 0.93)
Total	0.35 (−0.08, 0.71)	0.93 (0.85, 0.97)	0.88 (0.72, 0.95)

The 95% confidence intervals are indicated in parenthesis

**Fig. 4:**
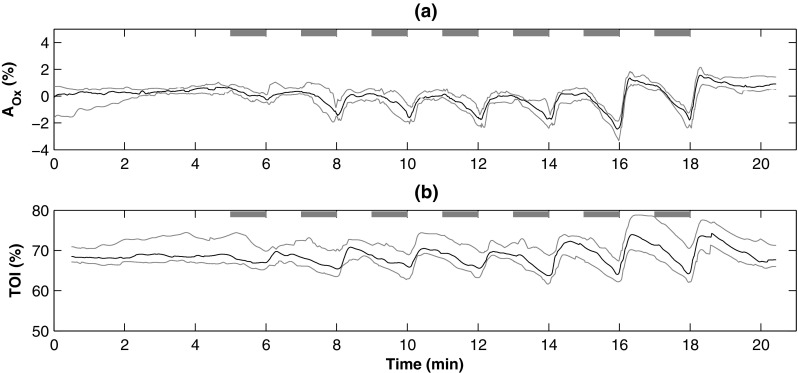
Changes in A$_{Ox}$ (**a**) and TOI (**b**) during the protocol across all the volunteers.* The black solid lines* represent the medians, while the* grey lines* are the upper (75%) and lower (25%) quartiles.* The grey horizontal bars* indicate the duration of the occlusions. The order of the occlusions was 20, 40, 60, 80, 100 mmHg, systolic pressure, and over-systolic pressure

Figure 4 shows the changes in oxygenation estimated from the DC PPG signals (A$_{Ox}$) throughout the protocol and the medians, upper, and lower quartiles across the population investigated. The changes in oxygenation estimated by the NIRS monitor (TOI) are also showed in the same figure with A$_{Ox}$ and the changes in the two oxygenation parameters are also summarised in Table 2. A$_{Ox}$ showed significant changes (p < 0.05) for all occlusions except for 20 mmHg (p = 0.06), while there were significant changes in TOI in all the different occlusions (p < 0.05). The same table presents the results of the correlation analysis between A$_{Ox}$ and TOI during the protocol, indicating a good correlation between the two parameters.

**Table 2:** Changes in A$_{Ox}$ during the occlusions in the protocol

Occlusions	A$_{{Ox}}$	TOI	${r}\,[{A}_{Ox}\,\text{vs. TOI}]$
20 mmHg	0.05 [−0.30 to 0.26]*	67.43 [65.81–72.24]	0.82 (0.61, 0.92)
40 mmHg	−0.65 [−1.38 to 0.64]	67.80 [64.32–71.10]	0.81 (0.60, 0.92)
60 mmHg	−0.59 [−1.54 to 0.18]	67.57 [64.17–71.54]	0.74 (0.46, 0.88)
80 mmHg	−1.03 [−1.25 to −0.24]	66.73 [64.83–72.53]	0.82 (0.61, 0.92)
100 mmHg	−1.11 [−1.68 to −0.26]	65.86 [63.73–70.26]	0.92 (0.83, 0.97)
Systolic	−1.72 [−2.22 to 1.43]	67.09 [64.65–70.51]	0.98 (0.95, 0.99)
Total	−0.95 [−1.56 to −0.51]	67.34 [64.54–72.79]	0.98 (0.96, 0.99)

The values were averaged over the last 30 s of each occlusion. The values are presented as medians, with the interquartile ranges in square brackets [(IQR = Q$_3$ − Q$_1$)]. The column on the right presents the correlation coefficients *r* between A$_{Ox}$ and TOI and the 95% confidence intervals within parenthesis

* Not significant changes from baseline (p > 0.05)

**Fig. 5:**
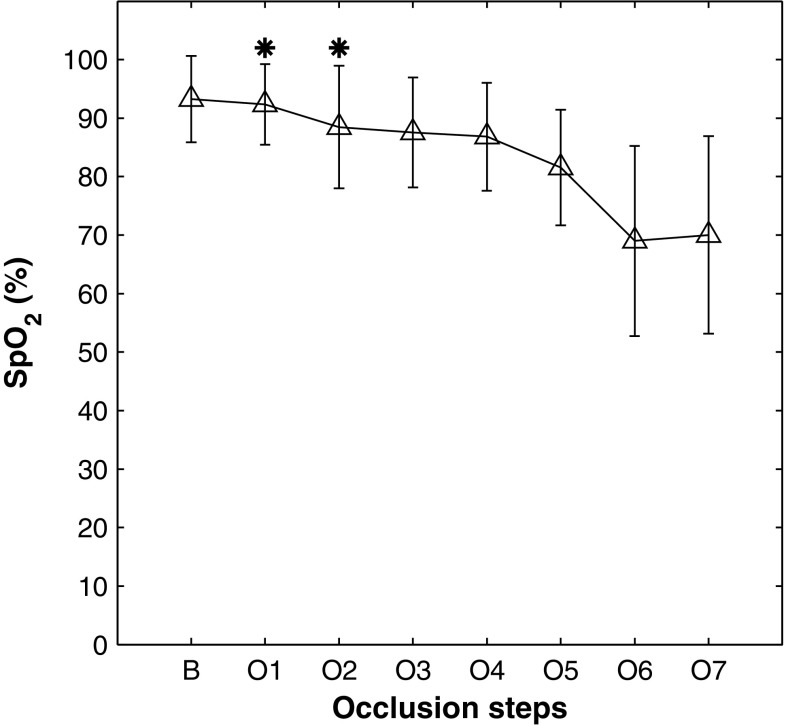
Mean changes in SpO$_2$ estimated from the forearm PPG during the protocol.* Errorbars* represent the standard deviation SD.* B* baseline, O1: 20 mmHg, O2: 40 mmHg, O3: 60 mmHg, O4: 80 mmHg, O5: 100 mmHg, O6: systolic pressure, O7: over-systolic pressure. Mean values were averaged over the last 30 s of each occlusion. Due to the absence of pulsatile signals in occlusions at the systolic and over-systolic pressure (O6 and O7), the estimated SpO$_2$ values could not be considered reliable. The* asterisks* indicate not significant changes from baseline (p > 0.05)

From the same PPG sensor on the forearm, the arterial oxygen saturation (SpO$_2$) was calculated from the ratio of the red and infrared PPG signals, as ordinarily performed in pulse oximetry. Figure 5 shows the mean changes in SpO$_2$ throughout the occlusions in the protocol. The changes in SpO$_2$ were not statistically significant in the partial venous occlusions (20–40 mmHg), whereas significant changes were observed in the following occlusions. For occlusion at the systolic and over-systolic pressure, the estimated values of SpO$_2$ are unreliable due to the absence of the pulsatile PPG signals.

## Discussion

The static DC component of the PPG signal in a pulse oximeter is often under-utilized when compared to the pulsatile AC component. However, it is well documented that this slowly varying component is a carrier of valuable physiological information [8–10] and changes in DC PPG during occlusion of the venous circulation have been reported firstly by Hertzman and Dillon [19], and later by Almond et al. [20]. Also, there is a growing interest in extracting new information from the PPG signal such as pulsatile pressure, venous oxygenation, or features of the PPG signal that could identify any other haemodynamic parameter different from SpO$_2$ [21–24]. This paper investigates further the feasibility of estimating relative changes in the concentration of oxygenated, reduced, and total haemoglobin from the DC component of the PPGs acquired from a custom-made, dual-wavelength reflectance sensor. The underlying hypothesis was that the DC component of the PPG signal might contain similar information as provided by Near Infrared Spectroscopy. Hence, by applying NIRS processing techniques to the DC PPG signal, the estimation of relative concentrations changes could be possible.

During the occlusion protocol in this study, the relative changes in haemoglobin concentrations determined from PPG measurements demonstrated to be responsive to all different occlusions, showing significant changes during occlusions of both venous and arterial nature. The protocol consisted of intermittent, pressure-increasing occlusions, which allowed analysing almost independently the response of the variables estimated. This also offered a different method compared to our previous protocol [17] and it allowed investigating the response of the PPG during partial venous occlusions (20–40 mmHg) and partial arterial occlusions (80–100 mmHg). Commonly, a change in perfusion can be recognised from NIRS signals by observing the trends in the relative changes in haemoglobin concentrations [5, 25]. For this reason, the directions of the changes were assessed by correlating PPG with simultaneous NIRS measurements. The results from this correlation analysis are very promising, but the low correlation of $\Delta$HbO$_2$ during over-systolic occlusions, which was likely to be caused by the short recovery period between occlusions, may require further analysis in the future. Although the relative changes in haemoglobin concentrations estimated from PPG showed good correlation and trending ability when compared with NIRS, some quantitative differences were still observed. These differences in the magnitude of the changes were assessed by Bland and Altman analysis of agreement and revealed a consistent underestimation of PPG compared to NIRS. However, differences in the magnitude of the changes between PPG and NIRS should be expected from the comparison of two systems with different sensors, emission wavelengths, the number of wavelengths, and sampled tissue volume.

Similar to the relative changes in haemoglobin concentrations, A$_{Ox}$ estimated from DC PPGs showed a responsivity to the occlusions that was comparable to the TOI measured by NIRS. The trends in the changes in A$_{Ox}$ were well correlated with TOI measurements and the mean values showed significant changes throughout the entire protocol. Differently from SpO$_2$ in pulse oximetry, which is estimated by isolating arterial blood via filtering the PPG signals, the changes in A$_{Ox}$ can be considered as a mixed contribution of venous and arterial components. This is because A$_{Ox}$ was obtained from mixed red and infrared DC PPG attenuations, without separating the arterial and venous components of the signals by any spectroscopic method. A similar and interesting approach to calculate oxygenation changes was previously applied by Nitzan et al. who used the DC component of the PPG signal, in conjunction with a venous occlusion, to estimate changes in venous oxygen saturations [26]. A$_{Ox}$, differently from TOI, can only be used as a mean for indicating trends changes rather than absolute oxygenation values. On this matter, because of the high variability between different NIRS devices and algorithms, the literature is more inclined in suggesting the use of TOI as a trending indicator instead of an absolute marker of oxygenation [6, 27, 28].

The results from the SpO$_2$ estimated from the forearm suggest that during low partial venous occlusions (20–40 mmHg), pulse oximetry was not able to detect a change. These findings are in line with other similar studies where the inadequacy of pulse oximetry to detect venous occlusion was reported [29–32]. The low values of SpO$_2$ from the forearm are in concordance with our previous results of forearm SpO$_2$ [17] and the high variability of SpO$_2$ from the wrist recently described by Harju et al. [33]. These results may suggest an increased presence of venous blood on the forearm, which is also supported by the changes in SpO$_2$ during venous congestion observed in this study and by the large respiratory component in the forearm PPG reported previously by Nilsson et al. [34].

The algorithm we used for estimating relative changes in haemoglobin concentrations from PPG (i.e. MBLL) enabled us to calculate the changes in the variables relative to the baseline, instead of absolute concentrations. The choice of the modified Beer-Lambert law to determine relative changes of $\Delta$HbO$_2$, $\Delta$HHb, and $\Delta$tHb was motivated by the easy application to dual-wavelength PPG signals, neither requiring major computational power nor additional instrumentation to current pulse oximetry technology.

It should be emphasised that NIRS, when compared to PPG, has an indisputable superiority in tissue penetration depth [5, 11]. Monitoring skin’s blood volumes and oxygenation can still have a significant clinical value [35], and the PPG may be adopted in situations when bulkier NIRS sensors cannot be employed and the monitoring of deep organs’ perfusion is not the main target.

Overall, the results from this study indicated that PPG could be applied in certain applications requiring the identification of venous or arterial occlusions. The most straightforward application may be in the monitoring of free flaps, where the prompt recognition of vascular complications such as venous congestions and arterial occlusions could improve the salvage rate and overall surgical outcomes [36]. Moreover, for this particular application, the PPG has the advantage of requiring a minimal surface, whereas NIRS usually requires a larger tissue area.

In conclusion, the results from this study demonstrated the capability of photoplethysmography in detecting changes in perfusion and oxygenation ($\Delta$HbO$_2$, $\Delta$HHb, $\Delta$tHb, and A$_{Ox}$) estimated from the DC PPGs during intermittent occlusions. The parameters, extracted from forearm PPG, showed good correlation and trending with simultaneous and local NIRS measurements. Estimating the relative changes in haemoglobin concentrations from the same PPG waveforms that are used to measure the SpO$_2$ and heart rate could provide and advantageous addition, with the possibility of extending further the range of applications of photoplethysmography.
